# Supplementary material for: An open-source framework for large-scale, flexible evaluation of biomedical text mining systems
Source: J Biomed Discov Collab. 2008 Jan 29;3:1. doi: 10.1186/1747-5333-3-1 (PMC2276192; doi:10.1186/1747-5333-3-1)
Supplement: Additional file 1 — Evaluation platform source code. The additional file contains the software evaluation framework discussed in this paper. The most current release can be downloaded from SourceForge.net [1]. See the accompanying README file for instructions on installation and use. Also included in the distribution is a collection of UIMA wrappers for some commonly used BioNLP tools and annotated corpora. [file 1747-5333-3-1-S1.zip › bionlp_uima_repository-1.0.1/docs/api/edu/uchsc/ccp/util/nlp/parser/corpus/package-summary.html]

edu.uchsc.ccp.util.nlp.parser.corpus (Center for Computational Pharmacology Util/UIMA API)


|  |  |  |
| --- | --- | --- |
| **Overview** \| **Package** \| Class \| **Tree** \| **Deprecated** \| **Index** \| **Help** | |  |
| **PREV PACKAGE**   **NEXT PACKAGE** | **FRAMES**    **NO FRAMES**     **All Classes** |  |


---

## Package edu.uchsc.ccp.util.nlp.parser.corpus

| **Class Summary** | |
| --- | --- |
| **Bio1CorpusParser** | A parser for the Bio1 corpus. |
| **BioIECorpusParser** | Parser for the BioIE corpus. |
| **CorpusParser** | This is an abstract class for a corpus parser. |
| **GeniaCorpusParser** | A simple parser for the GENIA corpus which utilizes the LingPipe utilities in order to extract entities and sentences from the corpus. |
| **IProLinkCorpus1Parser** | A parser for the iProLink corpus1.xml file. |
| **IProLinkCorpus2Parser** | A parser for the iProLink corpus2.xml file. |
| **IProLinkParser** | This class contains methods to parse the 2 IProLink XML corpora files. |
| **TexasCorpusParser** | A parser for the Texas corpus. |
| **YapexCorpusParser** | This class is used to parse out the gold standard annotations from the Yapex corpus.    Output Classes:  protein |

---


|  |  |  |
| --- | --- | --- |
| **Overview** \| **Package** \| Class \| **Tree** \| **Deprecated** \| **Index** \| **Help** | |  |
| **PREV PACKAGE**   **NEXT PACKAGE** | **FRAMES**    **NO FRAMES**     **All Classes** |  |


---
